# CD126^hi^ umbilical cord mesenchymal stem cells sensitive to IL-6 ameliorate inflammatory bowel disease by producing TGF-β1

**DOI:** 10.1186/s13023-025-03993-w

**Published:** 2025-08-27

**Authors:** Yanxia Fu, Bingchen Xie, Yinyin Wang, Jianqiu Sheng, Zhijie Chang, Xiaojue Qiu, Dongliang Yu, Junfeng Xu

**Affiliations:** 1 Department of Biochemistry and Molecular Biology, Capital Medical University, Beijing, 100069 China; 2 School of Biomedical engineering, Capital Medical University, Beijing, 100069 China; 3 State Key Laboratory of Membrane Biology, School of Basic Medical Sciences, Institute of Precision Medicine, Tsinghua University, Beijing, 100084 China; 4 Department of Gastroenterology, The Seventh Medical Center of Chinese PLA General Hospital, Beijing, 100700 China; 5 Department of Gastroenterology, The First Medical Center of Chinese PLA General Hospital, No.28 Fuxing Road, Haidian District, Beijing, 100853 China

**Keywords:** Inflammatory bowel disease, Human umbilical cord mesenchymal stem cells, Inflammation, T helper cells

## Abstract

**Background:**

Human umbilical cord mesenchymal stem cells (HUMSCs) are effective therapies for inflammatory bowel disease. However, the mechanisms remain unresolved. We found HUMSCs express CD126 (IL-6 receptor), which indicated CD126 sub-populations might show a distinct response to inflammation. In the present study, we explored whether CD126 is a critical molecule for HUMSCs in regulating inflammation.

**Methods:**

We assessed the regulatory effects of CD126 high (CD126^hi^) on the T lymphocyte subpopulations and related cytokines in the dextran sulfate sodium (DSS)-induced colitis model. The effect of CD126^hi^ was evaluated by Hematoxylin and Eosin (H&E) staining, fluorescence-activated cell sorting (FACS), and enzyme-linked immunosorbent assay (ELISA) analyses. Statistical significance was typically determined using Student’s t-test or one-way analysis of variance (ANOVA) with Tukey test.

**Results:**

The disease symptoms were markedly ameliorated and the interleukin-6 (IL-6), interleukin-17 (IL-17), interferon-γ (IFN-γ), Tumor necrosis factor-α (TNF-α), and interleukin-4 (IL-4) levels were significantly reduced in DSS-treated mice administered with CD126^hi^ HUMSCs but not in DSS-treated mice administered with CD126 low (CD126^lo^) HUMSCs. Intriguingly, CD126^hi^ HUMSCs significantly increased the levels of transforming growth factor-β (TGF-β1) and interleukin-10 (IL-10) in DSS-treated mice, accompanied by an increase in regulatory T cells (Treg cells). In vitro experiments showed that CD126^hi^ HUMSCs secreted TGF-β1 in response to IL-6 stimulation, while CD126^lo^ HUMSCs were latent in the inflammatory environment. We considered that TGF-β1 secreted by CD126^hi^ HUMSCs regulated the balance of Treg cells and thus promoted the recovery of murine colitis.

**Conclusion:**

Our results revealed a mechanism wherein CD126^hi^ HUMSCs function as inflammatory sensors and secrete anti-inflammatory cytokines to rebalance the population of T cells. This study shed light on the potential therapeutic application of CD126^hi^ HUMSCs for inflammatory diseases such as inflammatory bowel disease.

## Background

Inflammatory bowel disease (IBD), including Crohn’s disease (CD) and ulcerative colitis (UC), is a multifactorial disease characterized by chronic inflammation of the intestine, abdominal pain, and diarrhea. The etiology of IBD is attributed to several factors [1]. Generally, it is believed that CD is associated with an abnormal Type 1 T helper cell (Th1 cell)-mediated response, whereas UC is associated with an atypical Th2 cell-mediated response [2]– [3]. In particular, UC is characterized by mucosal T-cell dysfunction, inflammatory cell infiltration, and abnormal production of proinflammatory cytokines [4].

Although the incidence rate of IBD is increasing worldwide, an adequate treatment approach remains to be developed. Therefore, novel therapeutic strategies, such as cell therapy, are required to improve treatment efficiency and reduce treatment-related side effects [5]. Therapies based on mesenchymal stem cells (MSCs) have provided convincing evidence for treating various inflammatory and autoimmune diseases with potential anti-inflammatory and immunomodulatory effects [6, 7]. Many studies have demonstrated that MSCs can effectively alleviate DSS-induced colitis in mice, systemic lupus erythematosus nephritis, and graft-versus-host disease [8–10]. Currently, MSCs have been used in more than 100 clinical trials worldwide to treat a wide range of diseases. Favorable data from clinical studies indicate that treatment of IBD patients with autologous or allogenic MSCs significantly reduced the disease activity index (DAI) and had no adverse effects [11].

Although MSCs show promising results and their safety has been confirmed, several issues regarding the mechanisms of MSCs after administration remain unresolved. A potential mechanism is that MSCs migrate to the inflammation site, where they regulate the function of immune cells [12]. Previous studies have shown that MSCs suppress macrophage activation [13]; regulate activation and proliferation of T lymphocytes (T cells), B lymphocytes (B cells), and natural killer cells (NK cells); inhibit dendritic cells (DC) maturation; and promote the generation of regulatory T cells (Treg cells) [14–17]. Treg cells, which are important in regulating immune responses by selectively suppressing effector T cells, are considered to play a critical role in maintaining gut homeostasis and limiting intestinal inflammation [18]. At the molecular level, MSCs reduce the levels of proinflammatory cytokines, including IFN-γ, TNF-α, IL-6, and IL-4, but increase the levels of anti-inflammatory cytokines such as TGF-β1 and IL-10. Interestingly, MSCs express CD119 (IFN-γ receptor), CD120 (TNF-α receptor), CD126 (IL-6 receptor), CD124 (IL-4 receptor), and LAP (TGF-β receptor) on their cell surface [19]. This finding indicated that MSCs might play a role in regulating inflammation through some inflammatory cytokine signaling pathways.

Through flow cytometry analysis, we found that HUMSCs expressed low level of CD120, CD124 and LAP, but the expression of CD126 was much higher, which indicated CD126 sub-populations might show a distinct response to inflammation. This can also explain why HUMSCs have dual effects of anti-inflammatory and immune regulation. In the present study, we explored whether CD126 is a critical molecule for MSCs in regulating inflammation. We found that CD126^hi^ HUMSCs show therapeutic potential for treating DSS-induced colitis in C57BL/6J mice.

## Materials and methods

### Animals

Healthy C57BL/6J mice were obtained from Vital River Laboratories (Beijing, China). The mice were maintained in a pathogen-free room and fed an autoclaved pellet diet and water *ad libitum*. All mice were housed in isolated ventilated cages (six mice per cage) with a barrier facility at Tsinghua University. The mice were maintained on a 12-h light/dark cycle at 22–26 °C with sterile pellet food and water *ad libitum*. The laboratory animal facility has been accredited by AAALAC (Association for Assessment and Accreditation of Laboratory Animal Care International), and the IACUC (Institutional Animal Care and Use Committee) of Tsinghua University approved all animal experimental protocols used in this study (No. 2020-3-20). All efforts were made to minimize the number of animals used in the experiments and to reduce their suffering.

### Cell cultures

Human umbilical cord mesenchymal stem cells (HUMSCs) were cultured in high-glucose Dulbecco’s modified Eagle’s medium (Gibco, Grand Island, NY) supplemented with 2 mM L-glutamine, 5% fetal bovine serum (FBS, Gibco), 100 U/mL penicillin and 100 µg/mL streptomycin (P/S, Gibco), and cytokines (epidermal growth factor, basic fibroblast growth factor, platelet-derived growth factor, and insulin-like growth factor) [5]. The cultures were maintained at 37 °C in a humidified atmosphere with 5% CO_2_. Adherent spindle-shaped cells were cultured to 80% confluence, trypsinized using 0.25% trypsin (Gibco, Grand Island, NY), and passaged in the medium as described above.

### Immunophenotypic analysis of HUMSCs

To determine the expression of classical MSC markers, the cells were analyzed by flow cytometry by using anti-human antibodies against CD44, CD45, CD90, CD105, HLA-DR (human leukocyte antigen-DR), and CD126 (supplied by eBioscience or Biolegend, USA).

### Colitis induction

Experimental colitis was induced by feeding mice with 3.5% (wt/vol) DSS (MP Biomedicals United States, Solon, OH, USA) in sterile drinking water for 6 days [5]. One day before DSS treatment, mice were randomly assigned to four groups (*n* = 6 per group). MSCs group was administered intravenously with 2 × 10^6^ CD126^hi^ or CD126^lo^ cells in 200 µL PBS per mouse (*n* = 6), DSS group was administered with 200 µL PBS alone (*n* = 6) on days 1, 3, and 5. Healthy mice fed with a normal diet and sterile water were used as the control group. Stool consistency, fecal bleeding, and weight loss were used to evaluate colitis severity [20]. The entire colon was removed, and its length was measured at the end of the experiment. Colonic tissues were then fixed with 4% formaldehyde overnight and then transferred to 75% ethanol. The tissues were then embedded in paraffin, and longitudinal sections of the entire colon were prepared for histological studies.

### In vivo evaluation of intestinal permeability

Intestinal permeability was assessed by fluorescein isothiocyanate (FITC)-labeled dextran method as reported previously [21]. Briefly, 6 days after DSS treatment, mice (*n* = 6 per group) were gavaged with FITC-dextran (molecular weight, 4000 Da; Sigma-Aldrich, Inc., St. Louis, MO, USA) at the concentration of 60 mg/100 g body weight. Blood was collected at 4 h after FITC-dextran gavage. The serum FITC-dextran level was measured with a fluorescence spectrophotometer (emission and excitation wavelengths: 485 and 530 nm, respectively).

### Measurement of cytokine levels

At the end of the experiment, mice were anesthetized with pentobarbital sodium, and their blood samples were collected (*n* = 6 per group). The serum levels of TNF-α, IFN-γ, IL-4, IL-17, IL-10, and TGF-β1 were detected using mice ELISA kits (eBioscience, San Diego, CA, USA) in accordance with the manufacturer’s instructions.

### Analysis of colonic macrophages

To isolate lymphocytes, the entire colon was taken out from the mice’s body, and the mesentery, Peyer’s patches, and fat content were removed. The colon was then placed in a medium (Roswell Park Memorial Institute [RPMI] 1640, 10% Fetal Bovine Serum [FBS], 1% Penicillin-Streptomycin [P/S] solution, 5 mM Ethylenediaminetetraacetic acid [EDTA], and 20 mM 4-(2-Hydroxyethyl)-1-piperazineethanesulfonic acid [HEPES]) and shaken in an incubator at 190 rpm and 37 °C for 30 min to wash off the epithelial cells. The remaining tissue was minced and digested with 10 U/mL collagenase CLISPA (Worthington Biochemical) and 0.1 mg/mL DNase I at 37 °C for 40 min. Subsequently, heavy-density cells were purified in 40% Percoll (Sigma, Inc., St. Louis, MO, USA) by centrifugation for 10 min at 800 *g*.

### Quantification of secreted factors

Trophic factors secreted by MSCs are considered the most likely mechanisms for their therapeutic activity [22]. TGF-β1 and IL-10 secretion by HUMSCs was assessed by commercial ELISA kits (eBioscience, San Diego, CA, USA) under the manufacturer’s instructions.

### RNA sequencing (RNA-seq) analysis

Differential genes were identified using the Voom algorithm in LIMMA package and EdgeR; after adjusting the false discovery rate, the genes were considered to show significantly differential expression if the adjusted *p*-value was < 0.05, with a > 2-fold change in the expression level. The differential genes were categorized into upregulated and downregulated gene sets and analyzed for significant pathway enrichment against the KEGG pathway database with an adjusted *p* value of < 0.05 using the GOseq tool.

### Statistical analysis

All the experiments were repeated at least three times. All data are expressed as mean ± SD. Differences between two groups or more than two groups were compared by Student’s t-test or one-way analysis of variance (ANOVA) with Tukey test, respectively. GraphPad Prism version 5.0 (GraphPad, San Diego, CA, USA) was used for statistical analysis. Differences were considered statistically significant at *p* < 0.05.

## Results

### Isolation and characterization of HUMSCs with high expression of CD126 (CD126^hi^) and low expression of CD126 (CD126^lo^)

**Fig. 1:**
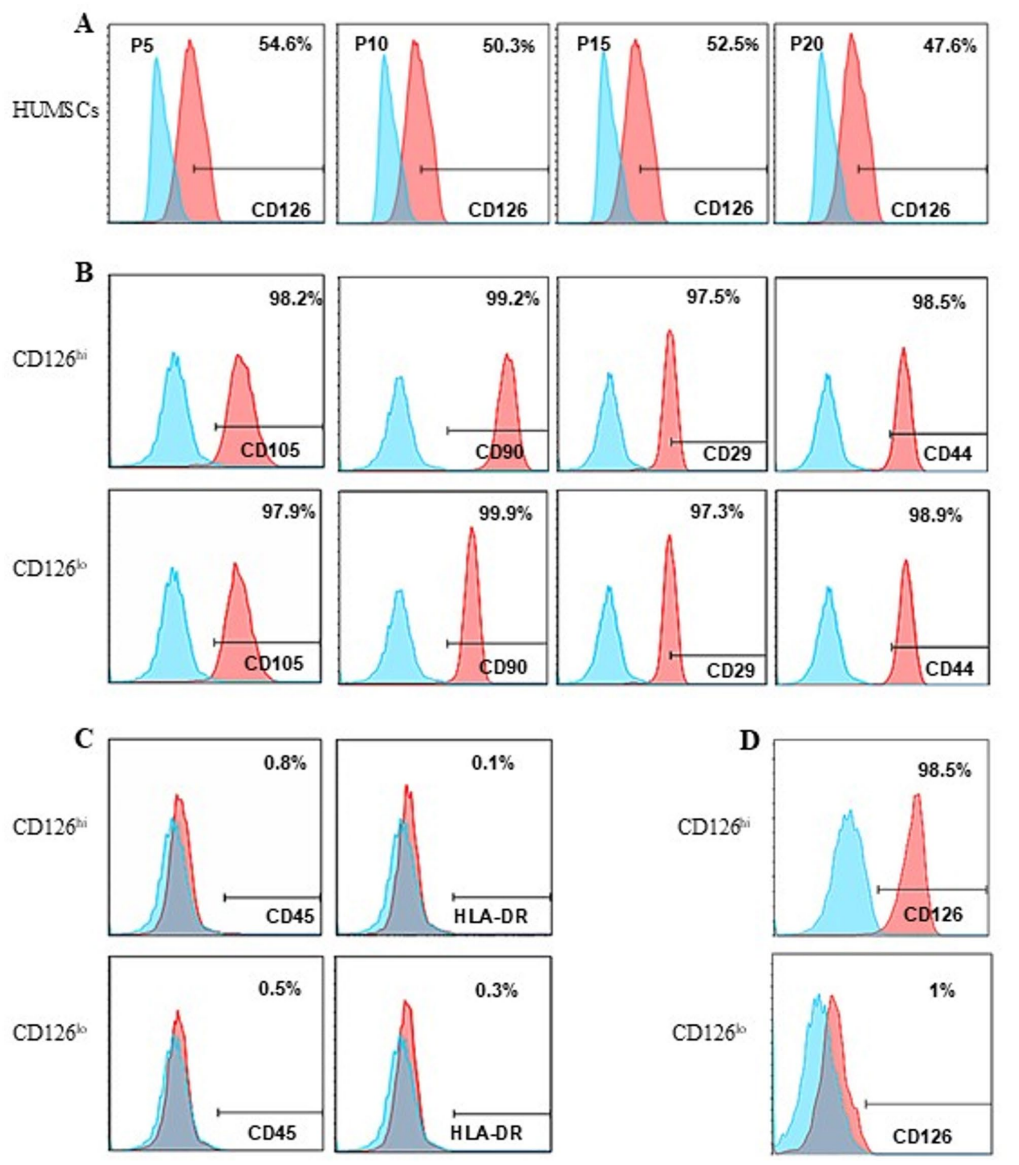
Flow cytometric analysis of cell surface markers of CD126^hi^ HUMSCs and CD126^lo^ HUMSCs. Flow cytometry was performed to determine the differences between CD126^hi^ HUMSCs and CD126^lo^ HUMSCs with regard to stem cell markers, leukocyte markers, and human leukocyte antigen-DR (HLA-DR). (**A**) CD126 expression levels in different generations of HUMSCs. (**B**) MSC markers expressed by CD126^hi^ HUMSCs and CD126^lo^ HUMSCs were detected by flow cytometry. (**C**) CD45 and HLA-DR expression levels in CD126^hi^ HUMSCs and CD126^lo^ HUMSCs. (**D**) CD126 expression level in CD126^hi^ HUMSCs and CD126^lo^ HUMSCs after flow cytometry

Human umbilical cord mesenchymal stem cells (HUMSCs) not only express stem cell markers but also express CD120 (TNF-α receptor), CD124 (IL-4 receptor), CD126 (IL-6 receptor) and LAP (TGF-β receptor). As the percent of CD120, CD124 and LAP was low (less than10%), but the percent of CD126 was much higher (about 50%), we speculated that CD126 sub-populations of HUMSCs might show a distinct response to inflammation. By performing flow cytometry analysis, we segregated CD126^hi^ HUMSCs from CD126^lo^ HUMSCs. Interestingly, CD126 expression remained constant in different generations of HUMSCs. In the 5th, 10th, 15th, and 20th generations, the expression of CD126 were 54.6%, 50.3%, 52.5% and 47.6%, respectively (Fig. 1A). Moreover, cells from these two subpopulations exhibited similar features of stemness as determined by the expression of markers CD105, CD90, CD29, and CD44 (Fig. 1B). Additionally, the expression rates of CD45 in CD126^hi^ and CD126^lo^ HUMSCs were 0.8% and 0.5%, respectively (Fig. 1C, the first panel). Both CD126^hi^ and CD126^lo^ HUMSCs showed almost no immune rejection features as the expression of HLA-DR was 0.1% and 0.3%, respectively (Fig. 1C, the last panel). After the cells were sorted using a fluorescent-activated cell sorter (FACS) with anti-IL-6R antibodies, we obtained almost unique populations of CD126^hi^ (98.5%) and CD126^lo^ (1%) HUMSCs (Fig. 1D).

### Effects of CD126^hi^ and CD126^lo^ HUMSCs on DSS-induced colitis

**Fig. 2:**
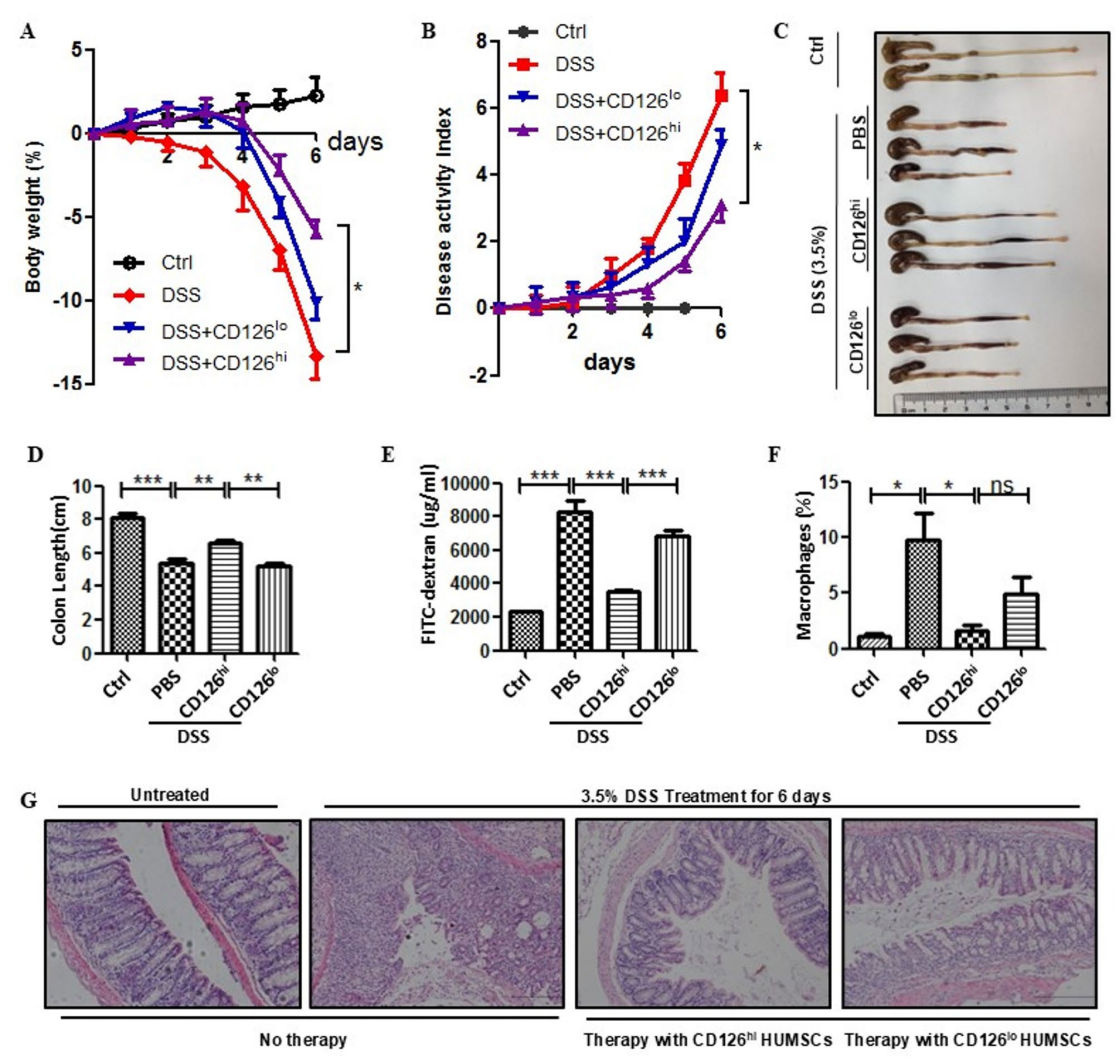
Effect of intravenous administration of CD126^hi^ HUMSCs and CD126^lo^ HUMSCs on DSS-induced colitis. Colitis was induced in mice by oral administration of 3.5% (wt/vol) DSS in sterile drinking water for 6 days (*n* = 6). Mice were intravenously administered 2 × 10^6^ cells in 200 µL PBS per mouse in the cell therapy group or 200 µL PBS per mouse in the DSS-treated group on days 1, 3, and 5. Error bars: mean ± SD. *p < 0.05; **p < 0.01; ***p < 0.005. (**A**) Body weight loss following DSS treatment. (**B**) Disease activity index (DAI) of colitis severity monitored based on weight change, stool consistency, and presence of fecal blood. (**C**) Measurement of the colon length. Representative colon image showing differences in the length of colons subjected to different treatments. (**D**) Statistical analysis of the colon length of the normal, DSS-treated, CD126^hi^ HUMSC-treated, and CD126^lo^ HUMSC-treated groups. (**E**) Evaluation of colon permeability based on the serum FITC-dextran level of colitis mice following the administration of CD126^hi^ HUMSCs or CD126^lo^ HUMSCs. (**F**) Percentage of macrophages in colonic tissues. (**G**) Histopathological analysis of the colon by hematoxylin-eosin (HE) staining. Scale bar = 100 mm

To compare the therapeutic effects of CD126^hi^ and CD126^lo^ HUMSCs on inflammation regulation, we used DSS-induced colitis mice treated with different HUMSCs (*n* = 6 per group). The intravenous administration of 2 × 10^6^ CD126^hi^ HUMSCs significantly prevented body weight loss (Fig. 2A, purple line) and reduced the DAI (Fig. 2B, purple line) in DSS-treated mice. In contrast, the administration of the same number of CD126^lo^ HUMSCs showed less effects on the recovery of body weight loss (Fig. 2A, blue line) and DAI reduction (Fig. 2B, blue line). Colonic structure analysis indicated that CD126^hi^ HUMSCs rescued the colon length shortened by DSS treatment; however, CD126^lo^ HUMSCs almost lost the ability to recover the colon length (Figs. 2C-D). CD126^hi^ HUMSCs, but not CD126^lo^ HUMSCs, drastically decreased colonic permeability (Fig. 2E). FACS analyses revealed that CD126^hi^ HUMSCs remarkably repressed macrophage proliferation in the colon; however, CD126^lo^ HUMSCs showed an impaired ability to reduce the number of macrophages (Fig. 2F). Consistent with alterations in colonic length and permeability, the histological structure of colonic crypts was destroyed by DSS treatment but almost recovered to the normal state by CD126^hi^ HUMSCs (Fig. 2G). However, the intravenous administration of the same dose of CD126^lo^ HUMSCs had little effect on the recovery of colonic crypts (Fig. 2G, last image). These results suggest that the therapeutic effects of HUMSCs on DSS-induced colitis may be mainly due to the presence of CD126^hi^ cells.

### CD126^hi^ HUMSCs alleviate DSS-induced colitis by regulating immune cells and inflammatory cytokines

**Fig. 3:**
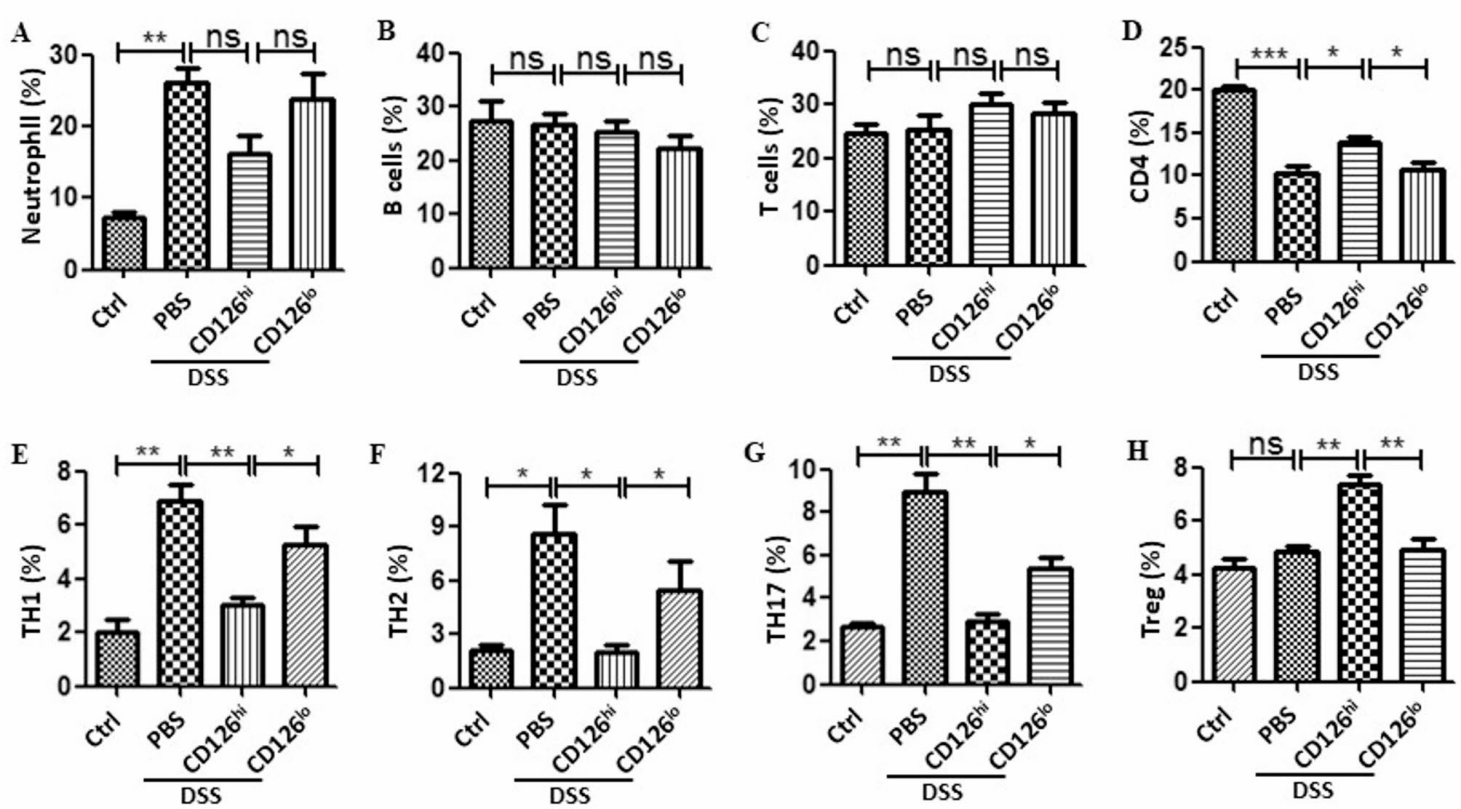
Different effects of CD126^hi^ HUMSCs and CD126^lo^ HUMSCs on immune cells and inflammatory cytokines in DSS-induced colitis mice. Colitis was induced in mice by oral administration of 3.5% (wt/vol) DSS in sterile drinking water for 6 days (*n* = 6). On the seventh day, the spleens of each group of mice were obtained and grounded into single cells; the cells were then cultured in RPMI 1640 medium containing 10% fetal bovine serum and 1% penicillin and streptomycin. The proportion of immune cells was detected by flow cytometry. Error bars: mean ± SD. *p < 0.05; **p < 0.01; ***p < 0.005. (**A-D**) Effect of CD126^hi^ HUMSCs and CD126^lo^ HUMSCs on the proportions of neutrophils, B cells, T cells, and T helper cells in the blood of DSS-induced colitis mice. (**E-H**) Effect of CD126^hi^ HUMSCs and CD126^lo^ HUMSCs on Th1, Th2, and Th17 cells and Treg cells in the spleens of DSS-induced colitis mice

To further understand how CD126^hi^ HUMSCs recovered DSS-induced colitis, we determined the effect of CD126^hi^ HUMSCs and CD126^lo^ HUMSCs on several immune cells, including neutrophils, B cells, T cells, T helper cells (Th1, Th2, and Th17 and Treg cells) by flow cytometry. No significant differences were observed in the percentage of blood neutrophils, B cells, and T cells (Figs. 3A-C); however, the proportion of CD4 was higher in CD126^hi^ HUMSCs-treated mice than in CD126^lo^ HUMSCs-treated mice (Fig. 3D). CD126^hi^ and CD126^lo^ HUMSCs decreased the percentages of Th1, Th2, and Th17 cells in the spleens of DSS-treated mice (Figs. 3E-G). However, mice treated with CD126^hi^ HUMSCs showed a lower proportion of Th17, Th2, and Th1 cells than those treated with CD126^lo^ HUMSCs (Fig. 3E-G, 3rd and 4th columns). We concluded that CD126^hi^ HUMSCs had a better effect on inhibiting the proliferation of proinflammatory T cells than CD126^lo^ HUMSCs. Additionally, the proportion of Tregs was significantly elevated by CD126^hi^ HUMSCs but not by CD126^lo^ HUMSCs (Fig. 3H). These findings suggest that the different therapeutic effects between CD126^hi^ and CD126^lo^ HUMSCs may result from their different effects on the regulation of T cell subsets.

**Fig. 4:**
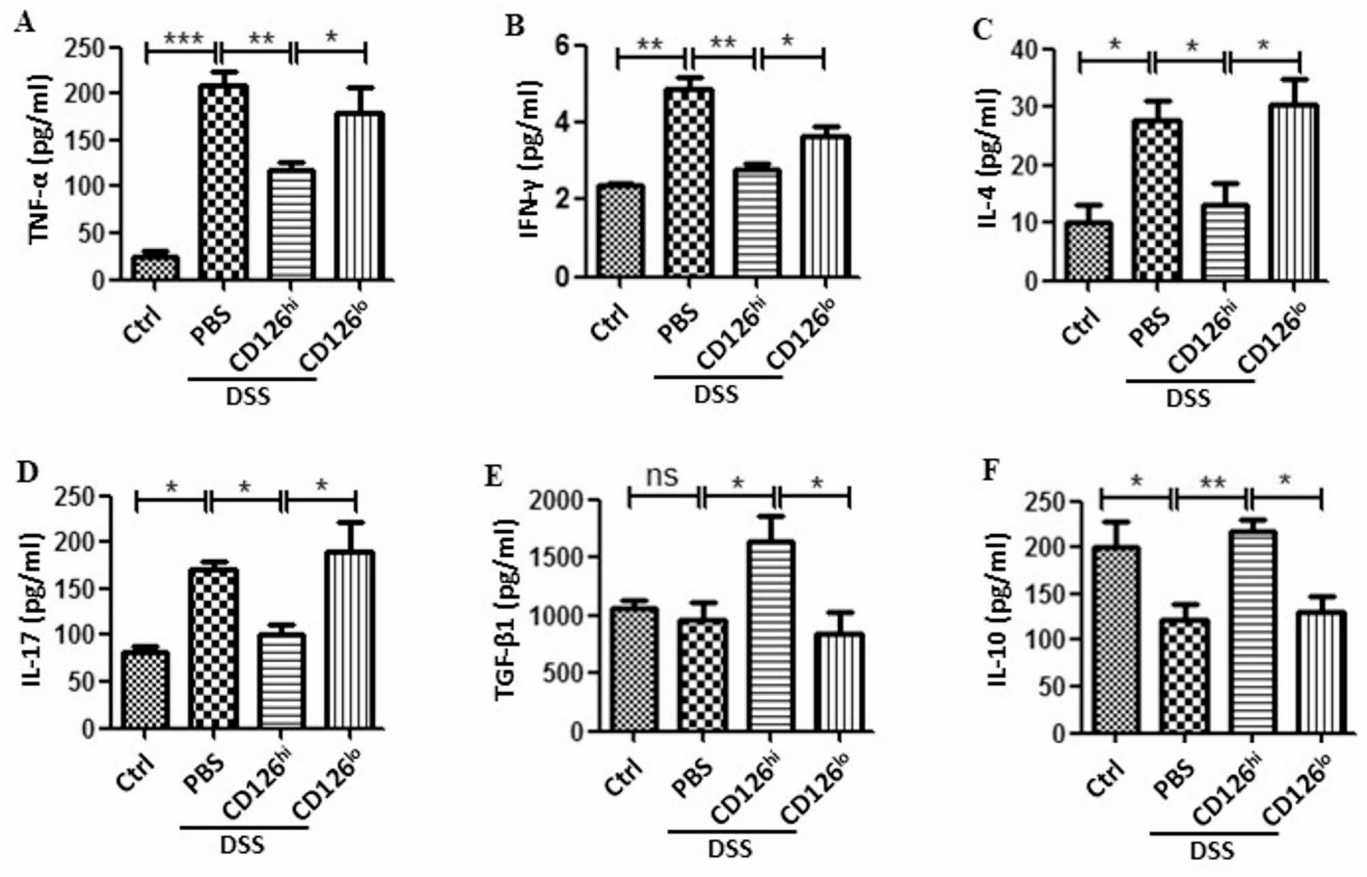
Different effects of CD126^hi^ HUMSCs and CD126^lo^ HUMSCs on inflammatory cytokines in DSS-induced colitis mice. (**A-D**) Serum TNF-α, IFN-γ, IL-4, and IL-17 levels in mice with or without MSC therapy were measured by ELISA (*n* = 6). (**E-F**) Serum levels of the anti-inflammatory cytokines TGF-β1 and IL-10 in mice with or without MSC therapy were measured by ELISA

We then investigated whether CD126^hi^ HUMSCs and CD126^lo^ HUMSCs showed different effects on the production of inflammatory cytokines in colitis mice (*n* = 6 per group). CD126^hi^ MSCs reduced TNF-α production induced by DSS; however, CD126^lo^ HUMSCs showed a retarded effect (Fig. 4A). Intriguingly, CD126^hi^ HUMSCs but not CD126^lo^ HUMSCs decreased the levels of IFN-γ, IL-4, and IL-17 (Figs. 4B-D). In contrast, TGF-β1 and IL-10 levels in DSS-treated mice were markedly increased by CD126^hi^ HUMSCs but not by CD126^lo^ HUMSCs (Figs. 4E-F). These results indicate that CD126^hi^ HUMSCs promote the production of anti-inflammatory cytokines and reduce the levels of proinflammatory cytokines.

Taken together, these results suggest that the anti-inflammatory effect of CD126^hi^ HUMSCs on DSS-induced colitis is mainly due to alterations in the proportions of macrophages and T cell populations and the production of their cytokines.

### Effect of CD126^hi^ HUMSCs on the proliferation and differentiation of macrophages and T cells

**Fig. 5:**
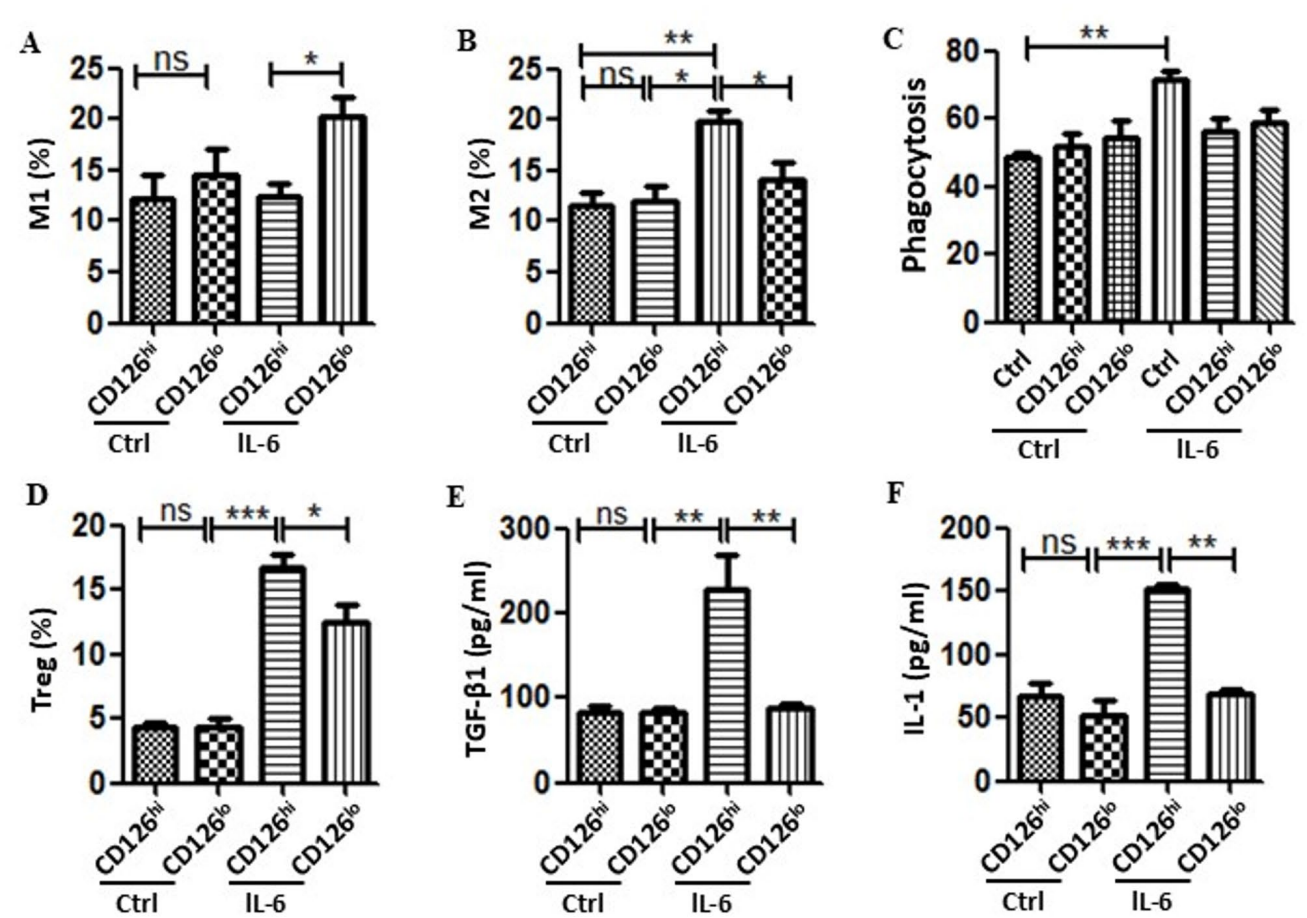
Effect of CD126^hi^ HUMSCs and CD126^lo^ HUMSCs on immune cell differentiation in vitro. (**A-B**) Effect of CD126^hi^ HUMSCs and CD126^lo^ HUMSCs on the differentiation of bone marrow-derived macrophages in the absence or presence of IL-6. (**C**) Effect of CD126^hi^ HUMSCs and CD126^lo^ HUMSCs on macrophage phagocytosis with or without IL-6 stimulation. The proportion of macrophages that phagocytosed FITC-dextran was considered to evaluate their phagocytic capacity. (**D**) Effect of CD126^hi^ HUMSCs and CD126^lo^ HUMSCs on the differentiation of Treg cells. (**E-F**) Effect of CD126^hi^ HUMSCs and CD126^lo^ HUMSCs on TGF-β1 and IL-10 secretion from induced Treg cells

To assess the role of CD126^hi^ HUMSCs and CD126^lo^ HUMSCs on both macrophage and T cell populations, we examined cell proliferation and differentiation ability in vitro. First, we determined the effect of HUMSCs on macrophages by co-culturing bone marrow-derived macrophages with CD126^hi^ HUMSCs and CD126^lo^ HUMSCs. CD126^hi^ HUMSCs strongly repressed M1 macrophages and promoted M2 macrophage differentiation when compared with CD126^lo^ HUMSCs in vitro (Figs. 5A-B). However, CD126^hi^ HUMSCs and CD126^lo^ HUMSCs with or without IL-6 stimulation showed no significant differences in the extent of macrophage phagocytosis (Fig. 5C).

Next, we examined the role of CD126^hi^ HUMSCs and CD126^lo^ HUMSCs in the proliferation and differentiation of T cells. CD126^hi^ HUMSCs and CD126^lo^ HUMSCs were co-cultured with spleen cells under IL-6 stimulation. Following IL-6 stimulation, CD126^hi^ HUMSCs increased the number of Treg cells as compared to CD126^lo^ HUMSCs (Fig. 5D). Consistent with this finding, under IL-6 stimulation, higher TGF-β1 and IL-10 levels were detected in the medium of spleen cells co-cultured with CD126^hi^ HUMSCs than in the medium of spleen cells co-cultured with CD126^lo^ HUMSCs (Figs. 5E-F). These observations suggest that CD126^hi^ HUMSCs regulate Treg cell differentiation in response to IL-6 stimulation.

### IL-6 stimulates CD126^hi^ HUMSCs to produce TGF-β1

**Fig. 6:**
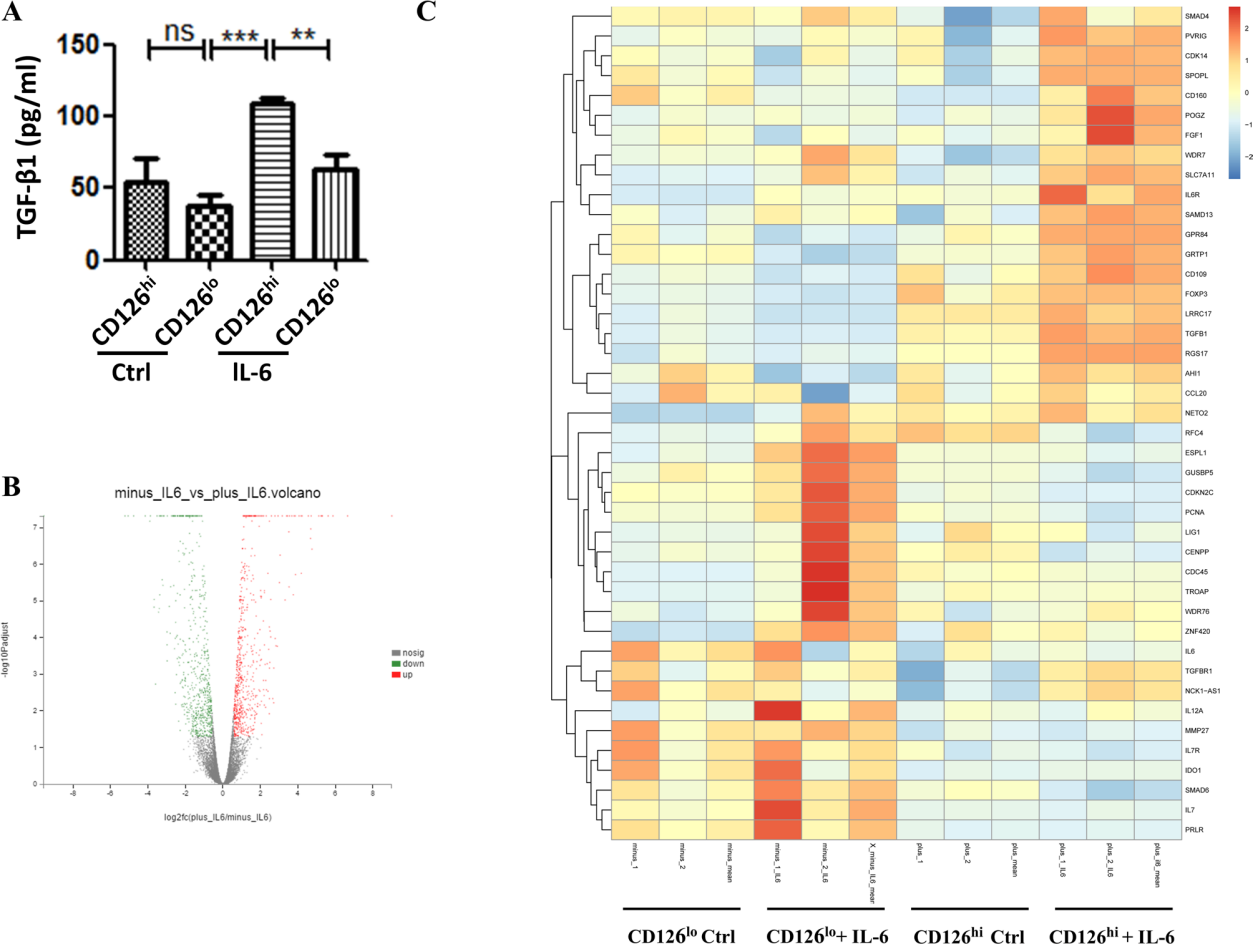
Mechanism of action through which CD126^hi^ HUMSCs alleviate colitis. (**A**) Difference in TGF-β1 secretion in the cell supernatant between CD126^hi^ HUMSCs and CD126^lo^ HUMSCs with or without IL-6 simulation. *p < 0.05; **p < 0.01; ***p < 0.005. (**B**) Volcano map of CD126^hi^ HUMSCs and CD126^lo^ HUMSCs stimulated with IL-6. (**C**) Sequencing analysis of CD126^hi^ HUMSCs and CD126^lo^ HUMSCs stimulated with or without IL-6

To determine the source of increased TGF-β1 level in DSS-challenged mice treated with CD126^hi^ HUMSCs, we stimulated both CD126^hi^ HUMSCs and CD126^lo^ HUMSCs with IL-6 in vitro. The results showed that CD126^hi^ HUMSCs and CD126^lo^ HUMSCs secreted equal amounts of TGF-β1 under normal conditions without IL-6 (Fig. 6A, see Ctrl). Interestingly, CD126^hi^ HUMSCs but not CD126^lo^ HUMSCs produced high levels of TGF-β1 under IL-6 stimulation. These results suggest that CD126^hi^ HUMSCs produce TGF-β1 in response to IL-6 stimulation, thus indicating that the elevated TGF-β1 level in DSS-induced colitis treated with CD126^hi^ HUMSCs originated from CD126^hi^ HUMSCs and Treg cells.

To analyze the other possible protein changes, we analyzed the gene expression profiles of CD126^hi^ HUMSCs and CD126^lo^ HUMSCs in response to IL-6. The RNA-seq data showed differential expression of several genes following IL-6 stimulation (Fig. 6B). Interestingly, the TGF-β1 mRNA level was increased in CD126^hi^ HUMSCs after IL-6 stimulation (Fig. 6C). We also observed changes in the expression patterns of several other genes. Among these, CD160, FGF, CD109, CCL20, and IL-6R were upregulated, while IL-6, IL-7, IL-12, and MMP-27 were downregulated (Fig. 6C). Taken together, these results demonstrated that CD126^hi^ HUMSCs express different cytokines following IL-6 stimulation to regulate the differentiation of macrophages and T cells.

## Discussion

**Fig. 7:**
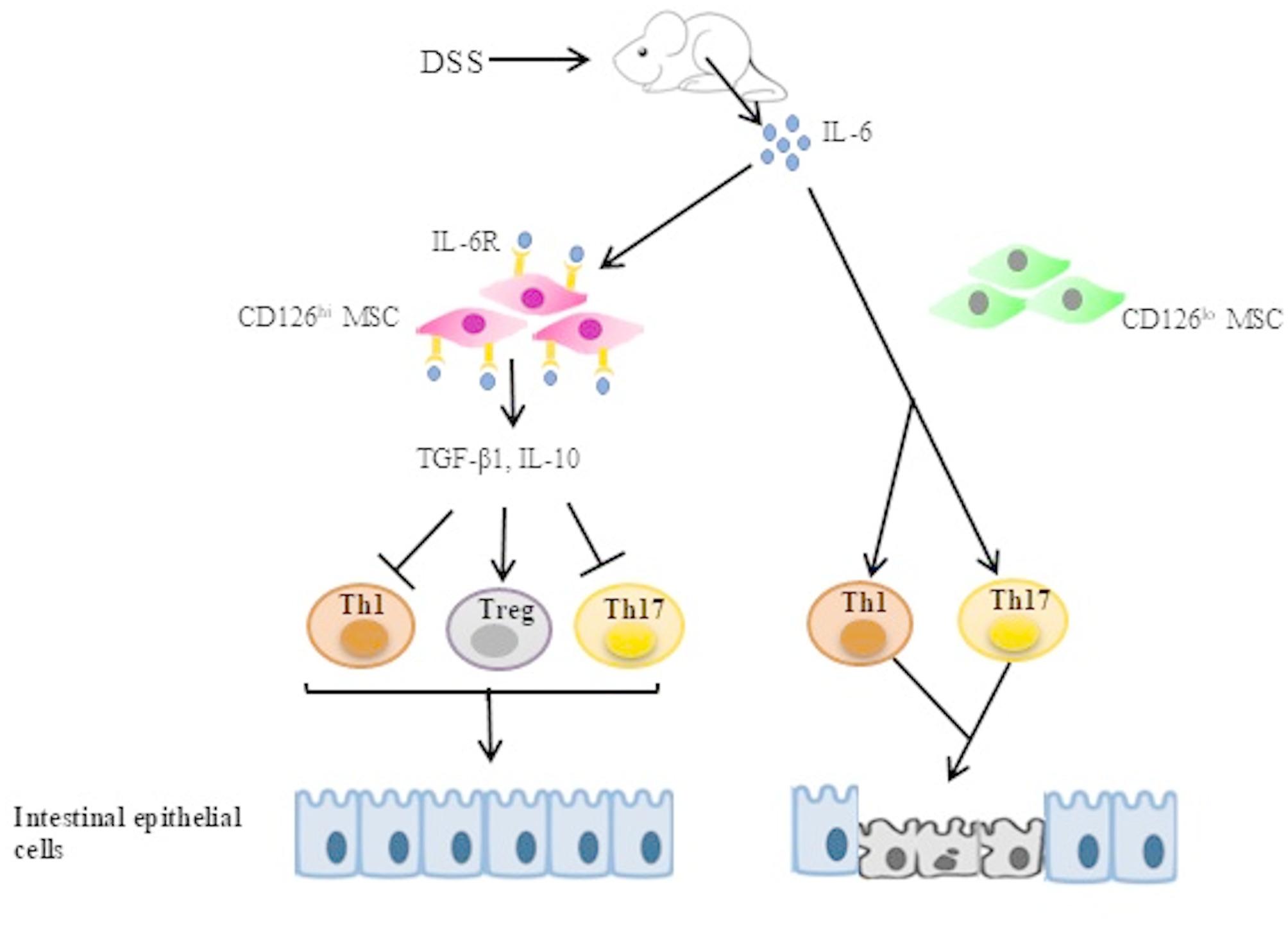
CD126^hi^ HUMSCs secrete TGF-β1 in response to IL-6 stimulation

In the present study, we elucidated that CD126^hi^ HUMSCs secrete TGF-β1 in response to IL-6 stimulation. Given that IL-6 is produced during inflammatory processes, CD126^hi^ HUMSCs respond to the elevated IL-6 level by generating negative regulators such as TGF-β1. This secretion of TGF-β1 subsequently modulates the imbalanced T cell population and regulates overactive macrophages. Based on our findings, we propose that CD126^hi^ HUMSCs sense the IL-6-dominant inflammatory environment. In contrast, in the presence of CD126^lo^ HUMSCs, IL-6 does not stimulate Th1 and Th17 cells during inflammation (Fig. 7). Thus, our study provides a new approach to repress inflammation with sensitive CD126^hi^ HUMSCs under the inflammatory environment. A noteworthy finding is that CD126^hi^ HUMSCs remain inactive under normal conditions without IL-6 stimulation. This feature is highly beneficial for treating inflammation-related diseases such as IBD, as CD126^hi^ HUMSCs may respond to different levels of inflammation depending on the IL-6 level.

Mesenchymal Stem Cells (MSCs) are positive for CD29, CD44, CD73, CD90, CD105, CD106, and CD126 markers and negative for hematopoietic lineage markers, including CD14, CD34, and CD45. To better understand the composition and phenotype of MSCs, it is useful to correlate the surface markers of MSCs with their bioactivity [23]. CD106 is critical for T cell activation and leukocyte recruitment to the inflammation site, and CD106-positive human term placental chorionic villi MSCs showed higher immunosuppressive activity than CD106-negative MSCs [24]. MSCs expressing high levels of CD146 enhanced suppressive properties in vitro by inhibiting alloreactive T cells in both soluble- and cell-contact-dependent manner [25]. CD166 can be used as a gene expression marker to characterize human mesenchymal stromal cells [26]. Because IL-6 is the predominant cytokine found in inflammatory tissues of UC patients [27], we assessed whether the IL-6 receptor could be a specific marker for HUMSCs. We found that CD126^hi^ HUMSCs but not CD126^lo^ HUMSCs were effective in alleviating IBD. We also observed that the intravenous administration of CD126^hi^ HUMSCs drastically decreased the DAI, regulated colonic permeability and body weight loss, and recovered damaged colonic structures. Therefore, we propose that the percentage of CD126^hi^ cells could be a marker of a better therapeutic effect on colitis.

DSS-induced colitis is a well-established animal model of mucosal inflammation for studying IBD pathogenesis. It is considered that DSS is directly toxic to gut epithelial cells and affects the integrity of the mucosal barrier [28]. Thus, DSS challenge leads to inflammation and further accelerates the damage of epithelial cells. The recovery of the damaged integrity of the mucosal barrier depends on the termination of inflammation. In the present study, we found that HUMSCs, in particular CD126^hi^ HUMSCs isolated from the umbilical cord, were effective in alleviating DSS-induced colitis. Treatment with CD126^hi^ HUMSCs increased colon length, prevented body weight loss, reduced colonic inflammatory cell infiltration, decreased proinflammatory cytokine production, and prolonged the survival of DSS-induced colitis mice. Although HUMSCs were observed to home at the damaged sites caused by DSS, their proportion was very low. Consequently, it was considered that the therapeutic effect of HUMSCs implanted into DSS-induced colitis mice was not due to cell homing but due to the regulation of immune cell ratio. Our results are consistent with previous clinical studies on the application of MSCs for treating IBD.

Although the precise etiology of IBD remains unclear, abnormal immune responses are considered to play a major role in IBD pathogenesis [29]– [30]. Th1, Th2, and Th17 cells and Treg cells play a key role in the development of experimental colitis [31–33]. Earlier studies have shown that MSCs exert their immunomodulatory function by inducing Tregs [34]. However, in the present study, we observed that CD126^hi^ HUMSCs not only increased the proportion of Tregs but also repressed Th1, Th2, and Th17 cells (Figs. 3E-G). Alterations in these cell populations were consistent with the levels of related cytokines, including INF-γ, IL-4, and IL-17 (Figs. 4B-D). Although Treg cells produce TGF-β, we found that CD126^hi^ HUMSCs caused elevation in Treg cell population to a lesser extent than the observed decrease in the proportion of Th1, Th2, and Th17 in DSS-treated mice. Moreover, the increment in the Treg cell population (Fig. 3H) was not proportional to the increase in TGF-β1 levels (Fig. 4F). We considered that the remarkable decrease in Th1, Th2, and Th17 cell populations might be due to the presence of TGF-β1 mainly produced by CD126^hi^ HUMSCs but not by Tregs. Taken together, we propose that CD126^hi^ HUMSCs balance the disordered immune cell populations by producing TGF-β1.

Macrophages are the central mediators of the innate immune system, and they play a key role in IBD [35]. Typically, macrophages are categorized as classically activated macrophages (M1) and alternatively activated macrophages (M2) [36]. M2 macrophages, characterized by the expression CD206, are immunosuppressive cells involved in Th2 cell activation and regulate extracellular matrix molecule synthesis, wound repair, and tumor progression [37]. In contrast, M1 macrophages are proinflammatory cells with the potent ability to promote Th1 cell activation and mediate acute inflammatory responses by producing proinflammatory cytokines such as TNF-α and IL-6 [38]. The proportions of M1 and M2 macrophages are increased and decreased, respectively, in colitis; this phenomenon is accompanied by induction of inflammatory cytokine production and anti-inflammatory cytokine suppression [39]. MSCs function as inflammatory sensors and can differentiate M1 macrophages from M2 macrophages in vitro [40].

## Conclusion

In the present study, we examined the ability of CD126^hi^ HUMSCs and CD126^lo^ HUMSCs to differentiate mouse bone marrow cells into macrophages. We found that CD126^hi^ HUMSCs promoted the differentiation of bone marrow cells into M2 macrophages. Therefore, macrophages could be one of the targets of CD126^hi^ MSCs for the effective treatment of IBD. Further studies are required to elucidate the detailed molecular mechanisms of CD126^hi^ MSCs in regulating macrophage activity.

## Data Availability

The data that support the findings of this study are available within the article or from the corresponding author upon reasonable request.

## Abbreviations

HUMSCs: Human umbilical cord mesenchymal stem cells
H&E: Hematoxylin and Eosin
FACS: Fluorescence-activated cell sorting
ELISA: Enzyme-linked immunosorbent assay
IL-6: Interleukin-6
IL-17: Interleukin-17
IFN-γ: Interferon-γ
TNF-α: Tumor necrosis factor-α
TGF-β1: Transforming growth factor-βCD: Crohn’s disease
UC: Ulcerative colitis
DAI: Disease activity index
DSS: Dextran sulfate sodium
Th1 cell: Type 1 T helper cell
FITC: Fluorescein isothiocyanate
HLA-DR: Human leukocyte antigen-DR
HUMSCs: Human umbilical cord mesenchymal stem cells
IBD: Inflammatory bowel disease
MSCs: Mesenchymal stem cells
Treg cells: Regulatory T cells
RPMI: Roswell Park Memorial Institute
FBS: Fetal Bovine Serum
P/S solution: Penicillin-Streptomycin solution
EDTA: Ethylenediaminetetraacetic acid
HEPES: 4-(2-Hydroxyethyl)-1-piperazineethanesulfonic acid

## References

[1] Wirtz S, Neufert C, Weigmann B, et al. Chemically induced mouse models of intestinal inflammation. Nat Protoc. 2007;2:541–6. 17406617 10.1038/nprot.2007.41

[2] Mannon PJ, Fuss IJ, Mayer L, et al. Anti-interleukin-12 antibody for active crohn’s disease. N Engl J Med. 2004;351:2069–79. 15537905 10.1056/NEJMoa033402

[3] Fu Y, Zhang C, Xie H, Wu Z, Tao Y, Wang Z, Gu M, Wei P, Lin S, Li R, He Y, Sheng J, Xu J, Wang J, Pan Y. Human umbilical cord mesenchymal stem cells alleviated TNBS-induced colitis in mice by restoring the balance of intestinal microbes and immunoregulation. Life Sci. 2023;334:122189. 37865178 10.1016/j.lfs.2023.122189

[4] Goncalves Fda C, Schneider N, Pinto FO, et al. Intravenous vs intraperitoneal mesenchymal stem cells administration: what is the best route for treating experimental colitis? World J Gastroenterol. 2014;20:18228–39. 25561790 10.3748/wjg.v20.i48.18228 PMC4277960

[5] Fu Y, Li J, Li M, Xu J, Rong Z, Ren F, Wang Y, Sheng J, Chang Z. Umbilical cord mesenchymal stem cells ameliorate Inflammation-Related tumorigenesis via modulating macrophages. Stem Cells Int. 2022;2022:1617229. 35694239 10.1155/2022/1617229 PMC9178412

[6] Abdolmohammadi K, Mahmoudi T, Alimohammadi M, Tahmasebi S, Zavvar M, Hashemi SM. Mesenchymal stem cell-based therapy as a new therapeutic approach for acute inflammation. Life Sci. 2023;312:121206. 36403645 10.1016/j.lfs.2022.121206

[7] Ha DH, Kim HK, Lee J, Kwon HH, Park GH, Yang SH, Jung JY, Choi H, Lee JH, Sung S, Yi YW, Cho BS. Mesenchymal stem/stromal Cell-Derived exosomes for Immunomodulatory therapeutics and skin regeneration. Cells. 2020;9(5):1157. 32392899 10.3390/cells9051157 PMC7290908

[8] Fu Y, Li J, Zhang Z, Ren F, Wang Y, Jia H, Liu J, Chang Z. Umbilical cord mesenchymal stem cell-derived exosomes alleviate collagen-induced arthritis by balancing the population of Th17 and regulatory T cells. FEBS Lett. 2022;596(20):2668–77. 35918178 10.1002/1873-3468.14460

[9] Li C, Wu F, Mao J, Wang Y, Zhu J, Hong K, Xie H, Zhou X, Tian J, Wen C. Mesenchymal stem cells-derived extracellular vesicles ameliorate lupus nephritis by regulating T and B cell responses. Stem Cell Res Ther. 2024;15(1):216. 39020448 10.1186/s13287-024-03834-w PMC11256400

[10] Yang H, Cheong S, He Y, Lu F. Mesenchymal stem cell-based therapy for autoimmune-related fibrotic skin diseases-systemic sclerosis and sclerodermatous graft-versus-host disease. Stem Cell Res Ther. 2023;14(1):372. 38111001 10.1186/s13287-023-03543-w PMC10729330

[11] Rodríguez-Fuentes DE, Fernández-Garza LE, Samia-Meza JA, Barrera-Barrera SA, Caplan AI, Barrera-Saldaña HA. Mesenchymal stem cells current clinical applications: A systematic review. Arch Med Res. 2021;52(1):93–101. 32977984 10.1016/j.arcmed.2020.08.006

[12] Mahmoudi M, Hoseinzadeh A, Rezaieyazdi Z, Afshari JT, Mahmoudi A, Heydari S. Cross talk between mesenchymal stem/stromal cells and innate immunocytes concerning lupus disease. Stem Cell Rev Rep. 2022;18(8):2781–96. 35876958 10.1007/s12015-022-10397-x

[13] Yin Y, Hao H, Cheng Y, Zang L, Liu J, Gao J, Xue J, Xie Z, Zhang Q, Han W, Mu Y. Human umbilical cord-derived mesenchymal stem cells direct macrophage polarization to alleviate pancreatic Islets dysfunction in type 2 diabetic mice. Cell Death Dis. 2018;9(7):760. 29988034 10.1038/s41419-018-0801-9 PMC6037817

[14] Gauthier BR, Rubio-Contreras D, Gómez-Rosado JC, Capitán-Morales LC, Hmadcha A, Soria B, Lachaud CC. Human omental mesothelial cells impart an Immunomodulatory landscape impeding B- and T-Cell activation. Int J Mol Sci. 2022;23(11):5924. 35682603 10.3390/ijms23115924 PMC9180401

[15] Cen W, Umrath F, Salgado AJ, Reinert S, Alexander D. Secretomes derived from osteogenically differentiated jaw periosteal cells inhibit phenotypic and functional maturation of CD14^+^ monocyte-derived dendritic cells. Front Immunol. 2023;13:1024509. 36700194 10.3389/fimmu.2022.1024509 PMC9868599

[16] Keating A. Mesenchymal stromal cells: new directions. Cell Stem Cell. 2012;10:709–16. 22704511 10.1016/j.stem.2012.05.015

[17] Murphy N, Treacy O, Lynch K, Morcos M, Lohan P, Howard L, Fahy G, Griffin MD, Ryan AE, Ritter T. TNF-α/IL-1β-licensed mesenchymal stromal cells promote corneal allograft survival *via* myeloid cell-mediated induction of Foxp3^+^ regulatory T cells in the lung. FASEB J. 2019;33(8):9404–21. 31108041 10.1096/fj.201900047R

[18] Tang RJ, Shen SN, Zhao XY, et al. Mesenchymal stem cells-regulated Treg cells suppress colitis-associated colorectal cancer. Stem Cell Res Ther. 2015;6:71. 25889203 10.1186/s13287-015-0055-8 PMC4414289

[19] Mark F, Pittenger AMM, Stephen C, Beck,Rama K, Jaiswal,Robin Douglas,Joseph D, Mosca,Mark A, Moorman,Donald W. Simonetti,Stewart craig,daniel R. Marshak. Multilineage potential of adult human mesenchymal stem cells. Science. 1999;284:143–7. 10102814 10.1126/science.284.5411.143

[20] Wang Y, Jiang X, Zhu J, et al. IL-21/IL-21R signaling suppresses intestinal inflammation induced by DSS through regulation of Th responses in lamina propria in mice. Sci Rep. 2016;6:31881. 27545302 10.1038/srep31881 PMC4992961

[21] Ibla JC, Khoury J. Methods to assess tissue permeability. Methods Mol Biol. 2013;1066:81–8. 23955735 10.1007/978-1-62703-604-7_7

[22] Xu J, Wang X, Chen J, Chen S, Li Z, Liu H, Bai Y, Zhi F. Embryonic stem cell-derived mesenchymal stem cells promote colon epithelial integrity and regeneration by elevating Circulating IGF-1 in colitis mice. Theranostics. 2020;10(26):12204–22. 33204338 10.7150/thno.47683 PMC7667691

[23] Mendicino M, Bailey AM, Wonnacott K, et al. MSC-based product characterization for clinical trials: an FDA perspective. Cell Stem Cell. 2014;14:141–5. 24506881 10.1016/j.stem.2014.01.013

[24] Yang ZXHZ-B, Ji YR, Wang YW, Liang L et al. CD106 identifies a subpopulat ion of mesench Ymal stem cells with unique Immu nomodulato Ry properties. PLoS ONE 2013;8. 10.1371/journal.pone.0059354 PMC3595282 23555021

[25] Bikorimana JP, Saad W, Abusarah J, Lahrichi M, Talbot S, Shammaa R, Rafei M. CD146 defines a mesenchymal stromal cell subpopulation with enhanced suppressive properties. Cells. 2022;11(15):2263. 35892560 10.3390/cells11152263 PMC9331786

[26] Brinkhof B, Zhang B, Cui Z, Ye H, Wang H. ALCAM (CD166) as a gene expression marker for human mesenchymal stromal cell characterisation. Gene X. 2020;5:100031. 32550557 10.1016/j.gene.2020.100031 PMC7285916

[27] Bernardo D, Vallejo-Diez S, Mann ER, et al. IL-6 promotes immune responses in human ulcerative colitis and induces a skin-homing phenotype in the dendritic cells and Tcells they stimulate. Eur J Immunol. 2012;42:1337–53. 22539302 10.1002/eji.201142327

[28] Riemschneider S, Hoffmann M, Slanina U, Weber K, Hauschildt S, Lehmann J. Indol-3-Carbinol and Quercetin ameliorate chronic DSS-Induced colitis in C57BL/6 mice by AhR-Mediated Anti-Inflammatory mechanisms. Int J Environ Res Public Health. 2021;18(5):2262. 33668818 10.3390/ijerph18052262 PMC7956562

[29] Haneishi Y, Furuya Y, Hasegawa M, Picarelli A, Rossi M, Miyamoto J. Inflammatory bowel diseases and gut microbiota. Int J Mol Sci. 2023;24(4):3817. 36835245 10.3390/ijms24043817 PMC9958622

[30] Geremia A, Biancheri P, Allan P, et al. Innate and adaptive immunity in inflammatory bowel disease. Autoimmun Rev. 2014;13:3–10. 23774107 10.1016/j.autrev.2013.06.004

[31] Huang C, Mei Q, Lou L, Huang Z, Fu Y, Fan J, Wang J, Yin N, Zheng Y, Lu Y, Zeng Y. Ulcerative colitis in response to fecal microbiota transplantation via modulation of gut microbiota and Th17/Treg cell balance. Cells. 2022;11(11):1851. 35681546 10.3390/cells11111851 PMC9180439

[32] Gomez-Bris R, Saez A, Herrero-Fernandez B, Rius C, Sanchez-Martinez H, Gonzalez-Granado JM. CD4 T-Cell subsets and the pathophysiology of inflammatory bowel disease. Int J Mol Sci. 2023;24(3):2696. 36769019 10.3390/ijms24032696 PMC9916759

[33] McLean LP, Cross RK, Shea-Donohue T. Combined Blockade of IL-17A and IL-17F May prevent the development of experimental colitis. Immunotherapy. 2013;5:923–5. 23998727 10.2217/imt.13.87 PMC3857957

[34] Mittal SK, Cho W, Elbasiony E, Guan Y, Foulsham W, Chauhan SK. Mesenchymal stem cells augment regulatory T cell function via CD80-mediated interactions and promote allograft survival. Am J Transpl. 2022;22(6):1564–77. 10.1111/ajt.17001 PMC11261724 35170213

[35] Zhang M, Li X, Zhang Q, Yang J, Liu G. Roles of macrophages on ulcerative colitis and colitis-associated colorectal cancer. Front Immunol. 2023;14:1103617. 37006260 10.3389/fimmu.2023.1103617 PMC10062481

[36] Lissner D, Schumann M, Batra A, et al. Monocyte and M1 Macrophage-induced barrier defect contributes to chronic intestinal inflammation in IBD. Inflamm Bowel Dis. 2015;21:1297–305. 25901973 10.1097/MIB.0000000000000384 PMC4450953

[37] Sica A, Mantovani A. Macrophage plasticity and polarization: in vivo veritas. J Clin Invest. 2012;122:787–95. 22378047 10.1172/JCI59643 PMC3287223

[38] Yunna C, Mengru H, Lei W, Weidong C. Macrophage M1/M2 polarization. Eur J Pharmacol. 2020;877:173090. 32234529 10.1016/j.ejphar.2020.173090

[39] Ebihara S, Urashima T, Amano W, Yamamura H, Konishi N. Macrophage polarization toward M1 phenotype in T cell transfer colitis model. BMC Gastroenterol. 2023;23(1):411. 38012544 10.1186/s12876-023-03054-1 PMC10680295

[40] Bernardo ME, Fibbe WE. Mesenchymal stromal cells: sensors and switchers of inflammation. Cell Stem Cell. 2013;13:392–402. 24094322 10.1016/j.stem.2013.09.006

